# Oral Supplementation of Lead-Intolerant Intestinal Microbes Protects Against Lead (Pb) Toxicity in Mice

**DOI:** 10.3389/fmicb.2019.03161

**Published:** 2020-01-22

**Authors:** Qixiao Zhai, Dingwu Qu, Saisai Feng, Yaqi Yu, Leilei Yu, Fengwei Tian, Jianxin Zhao, Hao Zhang, Wei Chen

**Affiliations:** ^1^State Key Laboratory of Food Science and Technology, Jiangnan University, Wuxi, China; ^2^School of Food Science and Technology, Jiangnan University, Wuxi, China; ^3^Wuxi Translational Medicine Research Center and Jiangsu Translational Medicine Research Institute, Wuxi, China; ^4^International Joint Research Laboratory for Probiotics, Jiangnan University, Wuxi, China; ^5^National Engineering Research Center for Functional Food, Wuxi, China; ^6^(Yangzhou) Institute of Food Biotechnology, Jiangnan University, Yangzhou, China; ^7^Beijing Innovation Center of Food Nutrition and Human Health, Beijing Technology and Business University, Beijing, China

**Keywords:** Pb toxicity, gut bacteria, intestinal barrier, oxidative stress, short chain fatty acids

## Abstract

Oral exposure to the heavy metal lead (Pb) causes various dysfunctions in animals. However, the influence of gut bacteria on Pb absorption, bioaccumulation, and excretion is largely unknown. In this study, we use a mouse model to investigate the relationship between gut microbiota, Pb-intolerant intestinal microbes and Pb toxicity. First, mice were treated with a broad-spectrum antibiotic cocktail to deplete their gut microbiota, and were then acutely and orally exposed to Pb at 1304 mg/kg for 3 days. Compared to the control mice, antibiotic-treated mice had increased Pb concentrations in the blood and primary organs and decreased Pb fecal concentrations, suggesting that gut microbiota limited the Pb burden that developed from acute oral Pb exposure. Next, three Pb-intolerant gut microbes, *Akkermansia muciniphila*, *Faecalibacterium prausnitzii*, and *Oscillibacter ruminantium*, were orally administered to mice, and their effects against Pb toxicity were evaluated. *F. prausnitzii* treatment significantly promoted the fecal Pb excretion and reduced Pb concentrations in blood (from 152.70 ± 25.62 μg/dL to 92.20 ± 24.33 μg/dL) and primary tissues. Supplementation with *O. ruminantium* significantly decreased Pb concentrations in blood (from 152.70 ± 25.62 μg/dL to 104.60 ± 29.85 μg/dL) and kidney (from 7.30 ± 1.08 μg/g to 5.64 ± 0.79 μg/g). Treatment with *F. prausnitzii* and *O. ruminantium* also upregulated tight junction (TJ) protein expression and the production of short-chain fatty acids by colonic microbiota, and showed protective effects against liver and kidney toxicity. These results indicate the potential for reducing Pb toxicity by the modulation of gut microbiota.

## Introduction

Lead (Pb), a potent environmental toxicant, is ubiquitous in daily life, and poses serious threats to public health. Pb may enter the body by ingestion or inhalation, and ultimately accumulates in blood, soft tissues, and bone. It has been reported that there is no safe threshold for Pb exposure and exposure to even low Pb levels (<10 μg/dL blood) can induce adverse health outcomes (Vorvolakos et al., 2016). The toxicity of Pb in the nervous, hematopoietic, hepatic, and renal systems has been well studied (Flora et al., 2012). However, the effect of Pb on the gut, one of the primary sites of absorption and excretion of metals (James et al., 1985), has received much less attention. To date, studies on the toxic effects of Pb on intestinal physiology have mainly focused on the following aspects. Histopathological observations have proved that Pb exposure can directly lead to local oxidative stress and inflammation in the gut, and caused marked intestinal morphological disorders (Crespo et al., 2006). Abnormal expression of genes such as divalent metal transporter 1 (DMT-1) and metallothioneins (MTs) was also observed in oral Pb-exposed mice, which resulted in impaired uptake of essential metals and other nutrients (Breton et al., 2013; Ebrahimi et al., 2015). Moreover, chronic Pb exposure can damage intestinal epithelial cells and tight junction (TJ) function, and perturbs the synthesis and secretion of intestinal mucin, ultimately disrupting the physical barrier of the gut (Holcombe et al., 1976; Breton et al., 2013).

The microbiota of the mammalian gut is recognized as crucial for the maintenance of the mammalian host’s health (Jandhyala et al., 2015). As oral ingestion is one of the main routes of Pb exposure, gut microbiota are inevitably exposed to this toxic metal. This damages the gut microbial community, as indicated by a decrease in the bacterial alpha diversity and a spatial shift of beta diversity (Wu et al., 2016). Recent reports have shown that both acute and chronic Pb toxicity induce dysbiosis of gut commensal organisms (Zhai et al., 2017; Xia et al., 2018). Metagenomic and metabolomic analysis revealed that some bacterial genera highly associated with the metabolism of energy, lipids, and bile acid (BA), including *Ruminococcus* and *Lachnospiraceae* species, are deleteriously affected by Pb exposure (Gao et al., 2017). Conversely, several enteric microorganisms such as *Enterococcus* (Topcu and Bulat, 2010) and sulfate-reducing bacteria such as *Desulfotomaculum acetoxidans* (Pado and Pawłowska-Cwiek, 2005) have been demonstrated to bind heavy metal ions such as Pb^2+^ *in vitro*. Furthermore, oral administration of probiotic intestinal bacteria, such as *L. plantarum* (Tian et al., 2012) and *Leuconostoc mesenteroides* (Yi et al., 2017), were observed to have protective effects against Pb toxicity in mice. Gut microbiota also play an essential role in maintaining intestinal homeostasis, protecting structural integrality of the gut mucosal barrier, and inhibiting the adverse effects of pathogens and xenobiotics on gut function (Jandhyala et al., 2015). However, little is known about the direct effect of whole-gut ecology on the absorption, distribution, and excretion of non-absorbed heavy metals. It is therefore necessary to study the interactions between intestinal microorganisms and Pb toxicity.

Previous studies yielded preliminary data on the effects of oral sub-chronic Pb exposure on the gut bacterial compositions of mice (Zhai et al., 2017). These studies showed that Pb exposure significantly decreased the abundances of microbial genera such as unclassified *Lachnospiraceae*, unclassified and uncultured *Ruminococcaceae*, *Oscillibacter*, *Anaerotruncus*, *Akkermansia*, *Lachnoclostridium Ruminiclostridium_9*, and *Rikenellaceae_RC9_gut_group*. These effects of were more drastic in the first month of treatment than in the last month. Among the altered microbial genera, the *Ruminococcaceae* are one of the main mucosa-associated microbe families in the human and murine colon (Nava and Stappenbeck, 2011). This family consists of several gut-resident bacteria such as *Faecalibacterium prausnitzii*, a commensal bacterium that has been reported to protect against immune disorders (Hornef and Pabst, 2015) and gut barrier hyperpermeability (Laval et al., 2015). In addition, the gut symbiont *Oscillibacter* has found to produce valeric or butyric acid (Takao et al., 2007; Lee et al., 2012) and *Oscillibacter* abundance is inversely associated with a wide range of diseases and dysfunctions, such as diarrhea and Crohn’s Disease (CD) (Wang et al., 2015; Fang et al., 2016). A range of studies have shown that *Akkermansia muciniphila*, a next-generation probiotic, plays an essential role in the regulation of host immune responses and metabolism (Cani and de Vos, 2017; Ottman et al., 2017). An outer membrane protein of the *A. muciniphila* strain Amuc_1100 has been shown to improve gut barrier functions by its interaction with Toll-like receptor 2 (TLR2) and by further activating the downstream NF-κB pathway (Plovier et al., 2017). In general, specific gut microbes may enhance the repair of intestinal mucosal wounds, maintain the homeostasis of gut immunity, and aid in the alleviation of gut inflammation. Consequently, we hypothesized that Pb exposure may damage the resident gut commensal bacteria, and thus disturb the structure and diversity of gut microbiota, which would further exacerbate heavy-metal absorption and gut barrier dysfunction. The direct oral administration of Pb-intolerant gut microbes may be of potential use for interventions against Pb toxicity.

We therefore assessed the role of the gut microbiota in the bioaccumulation and retention of Pb in mice, following oral Pb exposure. In addition, three Pb-intolerant gut microbes (*A. muciniphila*, *F. prausnitzii*, and *Oscillibacter ruminantium*) were administered to mice, and their effects against Pb toxicity were assessed.

## Materials and Methods

### Bacterial Strains and Culture

*Akkermansia muciniphila* ATTC BAA-835 was obtained from the American Type Culture Collection. *F. prausnitzii* A2-165 and *O. ruminantium* GH1 were obtained from the Japan Collection of Microorganisms. Other strains were obtained from the Culture Collections of Food Microbiology, Jiangnan University (Wuxi, China). All strains were cultured in de Man, Rogosa and Sharpe agar, Luria-Bertani broth, or Brain Heart Infusion medium (Hopebio Company, Qingdao, China) at 37°C. The cultured biomass was preserved immediately with 30% glycerol as the protectant. Colony counting was conducted to confirm the survival of bacteria for animal treatment.

### Animal and Experimental Diet

Six-week-old male C57BL/6 mice were purchased from the Shanghai Laboratory Animal Center (Shanghai, China). The mice had free access to standard commercial mouse chow and drinking water. At the end of the experiment, the mice were sacrificed by CO_2_ asphyxiation. Livers, kidneys, and other tissues were stored at −80°C prior to analysis. The protocols in this study were approved by the Ethics Committee of Jiangnan University, China (JN No. 20180615-c0801-20-2 and JN No. 20181030-c0301-11-0). All procedures in the study were carried out in accordance with European Community guidelines (Directive 2010/63/EU).

### Animal Experimental Design I

Forty mice were randomly divided into four groups (Table 1). In the first 7 days, mice were orally administered a combination of four antibiotics via oral gavage to deplete gut flora (Curtis et al., 2014). Feces were collected before and after antibiotic treatment to confirm depletion of the intestinal microbiota. In the last 3 days, four antibiotics were added to the drinking water and the mice were orally gavaged once daily with a dose of 1304 mg Pb acetate trihydrate (PbAc) per kg body weight. The dose was equivalent to one-fifth of the LD_50_ of Pb in mice (El-Ghor et al., 2011; Sun et al., 2017). Our preliminary experiment showed that this dose was non-lethal but exhibited a significant toxic effect in mice.

**TABLE 1:** The protocol of animal experimental design I.

Group	Experimental protocol	
	1–7 days	8–10 days
Control	SL	SL + PW
Antibiotics	AN-OG	SL + AN-PW
Pb	SL	PbAc-SL + PW
Antibiotics + Pb	AN-OG	PbAc-SL + AN-PW

*SL, 0.2 mL saline once daily via oral gavage; PW, plain water for drinking; AN-OG, four antibiotics (each dosed at 25 g/L) in 0.2 mL saline once daily via oral gavage; PbAc-SL, 30.4 g PbAc in 0.2 mL saline once daily via gavage; AN-PW, four antibiotics (ampicillin, neomycin, and metronidazole: 1 g/L; vancomycin: 500 mg/L) in drinking water.*

### Animal Experimental Design II

For chronic Pb exposure experiments, sixty mice were randomly divided into six groups (Table 2), mice in model group were exposed to Pb in their drinking water (at a dose of 1.83 g PbAc per L of drinking water), as described previously (Tian et al., 2012). All strains were provided as a dose of 1 × 10^8^ CFU once daily by oral gavage (Feng et al., 2019). Feces were collected every 2 weeks for the determination of Pb concentrations or for microbial DNA extraction.

**TABLE 2:** The protocol of animal experimental design II.

Group	Experimental protocol
	0–8 weeks
Control	SL + glycerol + PW
Pb	SL + glycerol + PbAc-PW
Pb + *L. plantarum*	SL + *L. plantarum* + PbAc-PW
Pb + *O. ruminantium*	SL + *O. ruminantium* + PbAc-PW
Pb + *F. prausnitzii*	SL + *F. prausnitzii* + PbAc-PW
Pb + *A. muciniphila*	SL + *A. muciniphila* + PbAc-PW

*SL + glycerol, 0.3 mL 3% glycerol solution (diluted with saline) once daily via gavage; PW, plain water for drinking; PbAc-PW, PbAc at 1.83 g/L in drinking water. SL + strains (*L. plantarum*, *O. ruminantium*, *F. prausnitzii*, and *A. muciniphila*), *L. plantarum* CCFM 8661, *A. muciniphila* ATCC BAA-835, *O. ruminantium* GH1, and *F. prausnitzii* A2-165 were preserved in 30% glycerol, −80°C. The bacterial suspension was diluted with 0.3 mL saline to a final concentration of 1 × 10^8^ CFU/0.3 mL and was administered once daily via oral gavage.*

### Pb Determination in Tissues, Blood, Urine and Feces

As described previously (Zhai et al., 2019), samples were digested by a microwave digestion system (MARS; CEM, United Kingdom). Pb concentrations in samples were subsequently quantified by atomic absorption spectrophotometry (Spectrum AAS or AA; Varian, United States).

### The qPCR-Based Determination of Total Bacterial Number

Microbial genomic DNA was isolated from feces and quantified by spectrophotometry. The primers for mouse genomic DNA were as previously described (Reikvam et al., 2011). The bacterial V3 region of the16S rRNA genes was amplified using primers 16S-F (5′-ACTCCTACGGGAGGCAGCAG-3′) and 16S-R (5′-ATTACCGCGGCTGCTGG-3′). The number of 16S DNA copies was normalized to the number of mouse genomic DNA copies for each sample. Threshold cycle values were used to calculate the number of 16S rRNA gene copies in each sample (Hill et al., 2010).

### Biochemical Analyses

The concentrations of malondialdehyde (MDA) and glutathione (GSH) in the tissues and the concentrations of alkaline phosphatase (ALP), aspartate transaminase (AST), urea, and creatinine (CREA) in plasma of mice were measured with assay kits purchased from the Jiancheng Bioengineering Institute (Nanjing, China).

### Histopathological Examination

The liver and kidney tissues were fixed in 10% formalin and embedded in paraffin. The tissue slices were subsequently stained with hematoxylin and eosin.

### Hematological Assays

The total white blood cell count (WBC), red blood cell count (RBC), eosinophil count, basophil count, mean corpuscular volume (MCV), lymphocyte count, hemoglobin and monocyte content, mean corpuscular hemoglobin (MCH) count, platelet hematocrit (PCT), mean corpuscular hemoglobin concentration (MCHC), and red blood cell distribution width (RDW) of each sample were analyzed with a fully automatic blood analyzer (BC-5000 Vet; Mindary, Shenzhen, China).

### The Evaluation of TJ Protein mRNA Expression in Intestinal Tissues

Total RNA was extracted with Trizol reagent and converted to cDNA using Taq-Man reverse transcription reagents (Applied Biosystems; Foster City, CA, United States). Real-time PCR parameters and primer sequences for the targeted mouse genes were as previously described (Zhai et al., 2018).

### The Determination of Intestinal Permeability in Mice

After fasting for 6 h, the mice were fed with DX-4000-FITC (sigma) at a dose of 600 mg/kg body weight. After 1 h, 100 mL of blood was taken from the tail vein and centrifuged at 3000 × *g* for 15 min. The supernatant was mixed with the same volume of PBS (pH 7.3), and the fluorescence intensity of the resulting solution was measured on a fluorescence microplate (SpectraMax; Molecular Devices, San Jose, CA, United States) (Cani et al., 2009).

### The Determination of Short-Chain Fatty Acids (SCFAs) in Feces

The concentration of SCFAs was measured by GC-MS (GCMS-QP2010 Ultra system, Shimadzu Corporation, Japan) (Wang et al., 2017). The concentration of total SCFAs was calculated as the sum of acetic, propionic, butyric, isobutyric, and pentanoic acid concentrations.

### The Determination of Pb-Binding Abilities of Specific Gut Microbes

As previously described (Halttunen et al., 2008), living cell pellets of tested strains were re-suspended in ultrapure water. containing 50 mg/L Pb^2+^, and the resulting solutions were incubated for 1 h. The residual Pb^2+^ concentrations were quantified by atomic absorption spectrophotometry (Spectrum AAS or AA; Varian, United States).

### The Determination of the Pb Tolerance of Specific Gut Microbes

The tolerance of the tested strains to Pb was determined by a Minimum Inhibitory Concentration (MIC) assay. PbAc solution was added to MRS or BHI liquid medium to give final Pb^2+^ concentrations of 0, 200, 250, 500, 1,000, 2,000, 3,000, 4,000, 5,000, and 6,000 mg/L. After inoculation of the activated bacterial liquid into the culture medium (to give a final concentration of 10^6^ CFU/mL), the Pb^2+^ concentration of each was measured three times. The lowest concentration of Pb^2+^ that could completely inhibit the growth of intestinal flora was taken to be the MIC (Youravong et al., 2011).

### Statistical Analysis

The data were represented as means ± SD. The Differences between each group were assessed by a one-way ANOVA test, followed by Tukey’s multiple-range tests. Statistical analyses and data visualization were conducted in GraphPad Prism (version 7; GraphPad Software Inc., La Jolla, CA, United States), unless otherwise stated.

## Results

### The Depletion of Gut Microbiota Enhanced Tissue Bioaccumulation of Pb in Acute Pb-Exposed Mice

Compared with control mice, both antibiotics-treated and antibiotics + Pb-treated mice lost weight dramatically and displayed significantly dilated caeca (Supplementary Figures S1A,B). Seven-day oral administration of antibiotics successfully depleted the gut commensals of mice, as indicated by treated mice having a three order of magnitude decrease in their gut microbiota compared to the controls (Supplementary Figure S1C). We also found that all mice treated with the antibiotics had markedly reduced copy numbers of 16S rRNA genes in feces, approximately 500 fold less than the levels in sham-treated mice (Supplementary Figure S1D).

**FIGURE 1:**
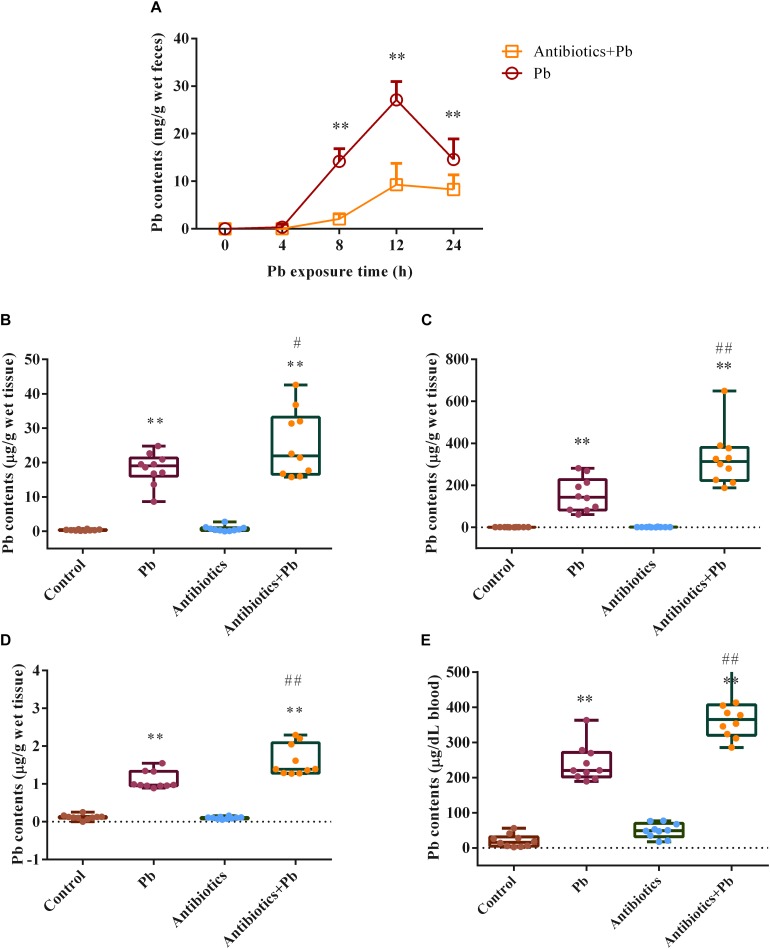
The effects of antibiotic treatment on Pb concentrations in feces, tissues and blood of mice (*n* = 10). **(A)**. Fecal Pb concentrations in the Pb-treated or Antibiotics + Pb-treated mice at different time points. **(B–E)**. Pb concentrations in the liver **(B)**, kidneys **(C)**, brain **(D)**, and blood **(E)** of mice. ^∗∗^*p* < 0.01 vs. the control group; ## *p* < 0.01 and #*p* < 0.05 vs. the Pb group.

Fecal Pb concentrations were markedly increased in the Pb-treated group (Figure 1A, *p* < 0.01) compared with the control mice, with fecal Pb concentrations in the control mice being too low to detect (data not shown). Combined antibiotic and Pb treatment significantly reduced fecal Pb concentrations at 8 h, 12 h, and 24 h (2.10 ± 1.10 mg/g, 9.28 ± 4.50 mg/g, and 8.29 ± 3.10 mg/g, respectively) compared with the group that received Pb alone (14.20 ± 2.66 mg/g, 27.10 ± 3.85 mg/g and 14.58 ± 4.34 mg/g, respectively) (Figure 1A, *p* < 0.01). Accordingly, antibiotic treatment enhanced Pb accumulation in the tissues and blood of the mice (Figures 1B–E).

### The Depletion of Gut Microbiota Aggravated the Disruption of Gut Barrier Function in the Acute Pb-Exposed Mice

**FIGURE 2:**
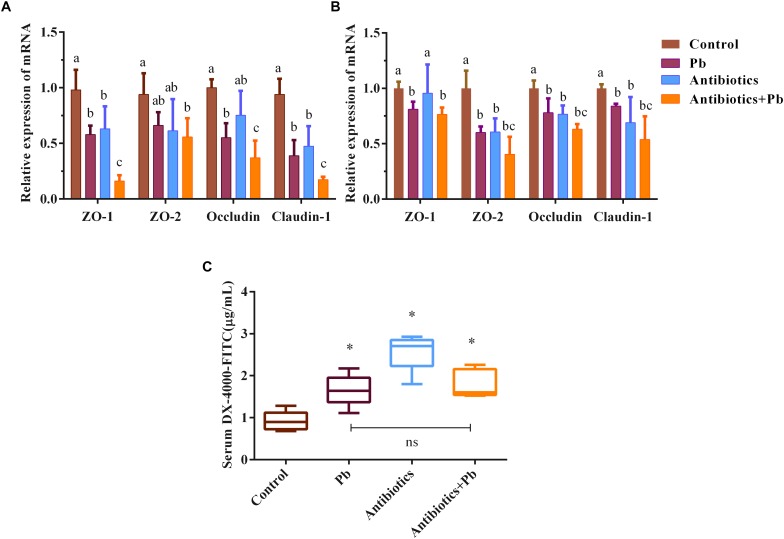
The effects of antibiotic treatment on the gut barrier function of mice (*n* = 5). mRNA expression of TJ proteins ZO-1, ZO-2, occludin and claudin-1 in representative sections of the colon **(A)** and small intestine **(B)** of mice. β-actin was used as the internal control. The data are expressed as the fold-change vs. control group (set to 1). Letters a–c denote statistically significant differences between each group (*p* < 0.05). **(C)** DX-4000-FITC levels in the serum of mice, ^∗^*p* < 0.05 vs. the control group. “ns” indicates no significant differences (*p* > 0.05) between groups.

As shown in Figure 2, acute Pb exposure led to increased gut permeability in mice, as indicated by the elevated concentrations of DX-4000-FITC in serum (from 0.92 ± 0.23 μg/mL to 1.66 ± 0.38 μg/mL, *p* < 0.05) and the decreased mRNA expression of ZO-1, ZO-2, occludin and claudin-1 genes in the colon and jejunum of mice (*p* < 0.05). Also, a greater decline was found in the relative expression of TJ mRNA in antibiotics-treated mice when compared with sham-treated counterparts (Figures 2A,B, *p* < 0.05).

### Oral Supplementation of Pb-Intolerant Intestinal Microbes Reduced Pb Burdens and Increased Fecal Pb Excretion in Chronically Pb Exposed-Mice

**FIGURE 3:**
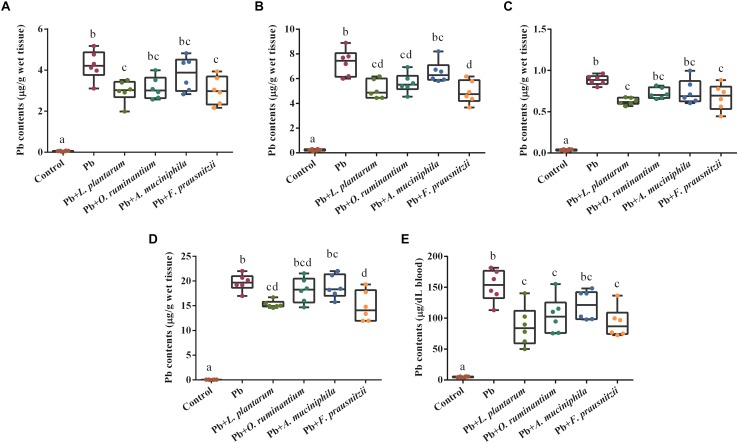
The effects of Pb-intolerant gut microbes on Pb concentrations in the liver **(A)**, kidneys **(B)**, brain **(C)**, small intestine **(D)**, and blood **(E)** of chronically Pb-exposed mice (*n* = 6). Letters a–d denote statistically significant differences between each group (*p* < 0.05).

Chronic Pb exposure induced higher concentrations of Pb in blood, liver, kidney and brain tissues of mice (Figure 3, *p* < 0.05). Oral supplementation of Pb-intolerant intestinal microbes, such as *F. prausnitzii* and *O. ruminantium*, markedly decreased Pb concentrations in the main target organs and blood of mice. However, administration of Pb-intolerant intestinal microbes did not restore the values of basic hematological parameters, except for HGB and MCH (Supplementary Tables S1, S2).

**FIGURE 4:**
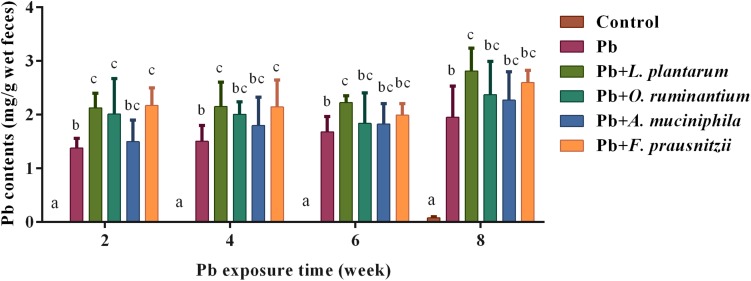
The effects of Pb-intolerant gut microbes on fecal Pb levels in chronically Pb-exposed mice at different time points (*n* = 6). Letters a–c denote statistically significant differences between each group (*p* < 0.05).

Oral supplementation of *F. prausnitzii* after 2 and 4 weeks of Pb exposure significantly promoted fecal Pb excretion (2.11 ± 0.31 mg/g and 2.15 ± 0.50 mg/g, respectively) (Figure 4, *p* < 0.05). *O. ruminantium* intervention also induced a higher fecal Pb content after the second week of Pb exposure (2.01 ± 0.66 mg/g) (Figure 4, *p* < 0.05).

### Oral Supplementation of Pb-Intolerant Intestinal Microbes Alleviated Hepatic and Renal Oxidative Stress in Chronically Pb-Exposed Mice

Although chronic Pb exposure induce no significant pathological lesion in mice (Supplementary Figures S2, S3), marked oxidative damage were observed in the livers and kidneys of the Pb-treated mice, indicated by their increased MDA levels (136.50 ± 25.32 mg/g and 119.80 ± 17.79 mg/g, respectively) and decreased GSH levels (0.96 ± 0.19 mg/g and 1.21 ± 0.22 mg/g, respectively) compared with the control mice (Table 3, *p* < 0.05). Similar to the effects of treatment with *L. plantarum*, *F. prausnitzii*, and *O. ruminantium* intervention markedly reduced MDA content in renal tissues (70.46 ± 23.11 mg/g and 76.88 ± 22.10 mg/g, respectively). However, there was no significant difference between the Pb-treated group and the Pb + *A. muciniphila*-treated group (Table 3, *p* > 0.05).

**TABLE 3:** The effects of Pb-intolerant gut microbes on the contents of MDA and GSH in the liver and kidneys of chronically Pb-exposed mice.

Group	Liver		Kidney	
	GSH (mg/g prot)	MDA (nmol/g prot)	GSH (mg/g prot)	MDA (nmol/g prot)
Control	4.68 ± 0.34^a^	40.73 ± 11.14^a^	5.01 ± 0.43^a^	39.28 ± 8.61^a^
Pb	0.96 ± 0.19^b^	136.50 ± 25.32^b^	1.21 ± 0.22^bd^	119.80 ± 17.79^b^
Pb + *L. plantarum*	1.98 ± 0.52^c^	84.21 ± 15.03^ac^	2.91 ± 0.78^c^	72.31 ± 14.32^ac^
Pb + *O. ruminantium*	1.51 ± 0.60^bc^	91.71 ± 31.98^b^	1.39 ± 0.65^bd^	76.88 ± 22.10^acd^
Pb + *A. muciniphila*	1.29 ± 0.34^bc^	97.58 ± 34.94^b^	1.06 ± 0.38^d^	114.44 ± 28.23^bd^
Pb + *F. prausnitzii*	1.90 ± 0.28^bc^	76.84 ± 25.17^ac^	2.15 ± 0.72^bc^	70.46 ± 23.11^ac^

*Data are expressed as means ± SD. Values are for six mice per group. Letters a–d indicate statistically significant differences (*p* < 0.05) between each group.*

Chronic Pb exposure significantly increased the concentrations of serum ALP, AST, UREA, and CREA in mice (Table 4, *p* < 0.05). All treatments with gut microbes, except for *A. muciniphila* treatment, restored these alterations. Compared with *L. plantarum* treatment, treatment with *F. prausnitzii* or *O. ruminantium* exhibited a similar protective effect on serum ALP, AST, UREA, and CREA.

**TABLE 4:** The effects of Pb-intolerant gut microbes on the values of haematogenic immunity parameters in chronically Pb- exposed mice.

Group	ALP (U/L)	AST (U/L)	UREA (mmol/L)	CREA-J (μmol/L)
Control	154.80 ± 7.46^a^	115.42 ± 8.76^a^	6.12 ± 0.50^a^	74.48 ± 3.97^a^
Pb	207.25 ± 19.14^b^	183.65 ± 18.71^b^	8.91 ± 0.79^b^	85.16 ± 3.40^b^
Pb + *L. plantarum*	166.00 ± 16.35^ac^	122.72 ± 12.56^cd^	676 ± 0.67^ac^	77.07 ± 4.57^ac^
Pb + *O. ruminantium*	176.38 ± 21.86^ab^	121.44 ± 19.08^c^	7.02 ± 1.20^ac^	79.97 ± 2.79^abc^
Pb + *A. muciniphila*	189.80 ± 17.75^bc^	153.59 ± 23.32^bd^	7.14 ± 0.70^ab^	81.53 ± 3.15^bc^
Pb + *F. prausnitzii*	170.00 ± 19.38^ac^	113.07 ± 14.57^c^	6.94 ± 1.08^ac^	79.86 ± 2.73^abc^

*Data are expressed as means ± SD. Values are for six mice per group. Letters a–d indicate statistically significant differences (*p* < 0.05) between each group.*

### Oral Supplementation of Pb-Intolerant Intestinal Microbes Relieved Intestinal Barrier Impairment Caused by Chronic Pb Exposure in Mice

**FIGURE 5:**
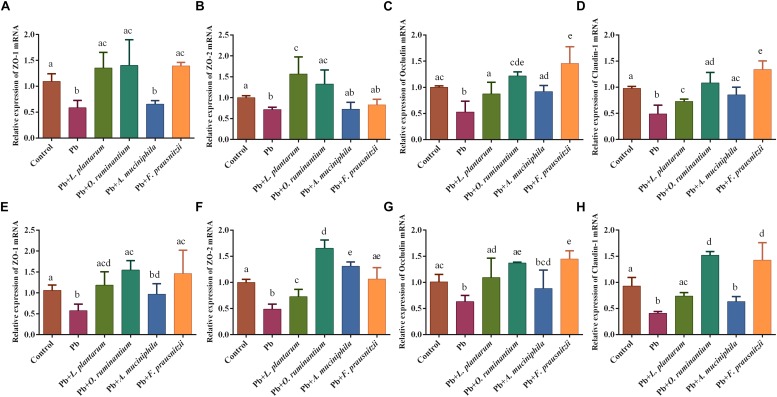
The effects of Pb-intolerant gut microbes on mRNA expression of TJ genes in representative sections of colon **(A–D)** and small intestine **(E–H)** of chronic Pb-exposed mice (*n* = 6). Letters a–e denote statistically significant differences between each group (*p* < 0.05).

The expression of TJ proteins in the colon and jejunum tissues of Pb-exposed mice decreased significantly compared to control mice (Figure 5, *p* < 0.05). Oral supplementation of *F. prausnitzii* or *O. ruminantium* significantly increased the expression of ZO-1, occludin and claudin-1 proteins in the colon and small intestine. Treatment with *A. muciniphila* had no influence on the expression of these TJ genes.

**FIGURE 6:**
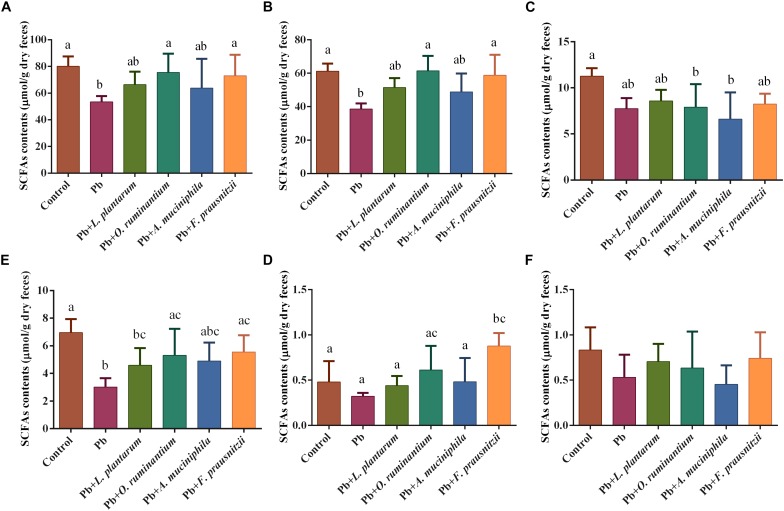
The effects of Pb-intolerant gut microbes on the total colonic contents of SCFAs **(A)**, acetic acid **(B)**, propionic acid **(C)**, butyric acid **(D)**, isobutyric acid **(E)**, and valeric acid **(F)** based on dry weights, of chronic Pb-exposed mice. Data are expressed as means ± SD (*n* = 6). Letter a–c refer to significant differences between each group (*p* < 0.05).

The levels of colonic gut bacterial metabolite SCFAs, such as acetic acid and butyric acid, were significantly reduced in the feces of Pb-exposed mice (61.26 ± 4.61 μmol/g and 3.01 ± 0.65 μmol/g, respectively) compared to the control group (38.54 ± 3.35 μmol/g and 6.95 ± 0.98 μmol/g, respectively) (Figure 6, *p* < 0.05). This reduction was reversed by the oral supplementation of Pb-intolerant intestinal microbes. Although a clear trend was observed, statistically significant differences were only found between the Pb-exposed group and *F. prausnitzii*- or *O. ruminantium-*treated groups for acetic acid and butyric acid levels and total SCFA content (Figure 6, *p* < 0.05).

### Pb-Binding and Pb-Sensitivity of Bacterial Strains

The Pb-sensitivity of the tested intestinal bacteria varied significantly (Table 5). When compared with *E. coli* K12, a resident bacterium in the gut, *F. prausnitzii* A2-165, *A. muciniphila* ATCC BAA-835, and *O. ruminantium* GH1 can be considered to be sensitive (intolerant) to Pb. Among the three Pb-intolerant gut microbes, *F. prausnitzii* A2-165 had the highest Pb tolerance, which is in close proximity to the Pb-tolerant strain.

**TABLE 5:** Pb tolerance abilities of the tested Pb-sensitive strains.

Strains	MIC (mg/L)
*L. plantarum* CCFM8661	5000
*O. ruminantium* GH1	200
*F. prausnitzii* A2-165	1000
*A. muciniphila* ATCC BAA-835	200
*E. coli* K12	1600

*The MIC for Pb of E. coli K12 has been reported by a previous study (doi:10.1016/j.ijantimicag.2007.03.012).*

**FIGURE 7:**
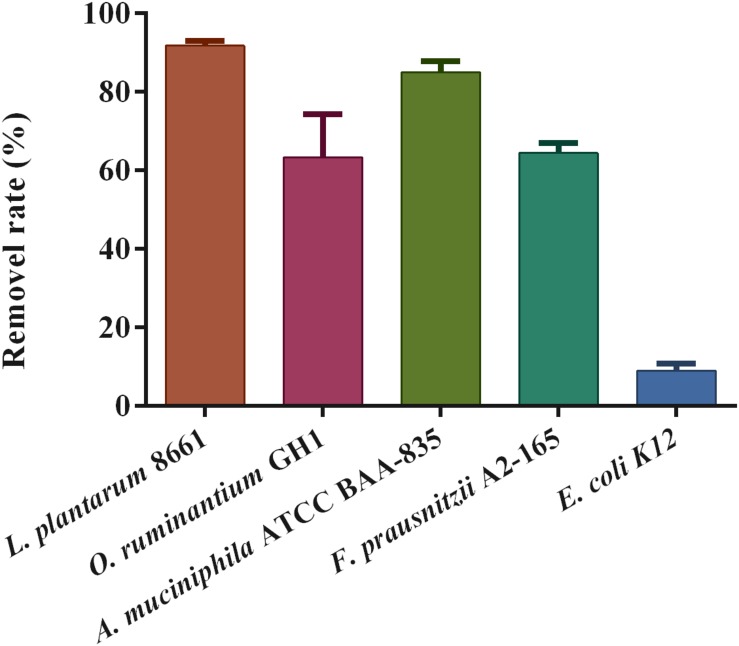
Pb-binding abilities of specific strains (*n* = 3).

The examination of the Pb removal rate showed that these strains have extensive differences in Pb-adsorption capacity, ranging from 9.02 ± 1.73% to 91.81 ± 1.16%. The three Pb-intolerant gut microbe strains showed a high adsorption capacity for Pb^2+^ compared with *E. coli* K12 (Figure 7). Among these, the binding capacity of *F. prausnitzii* A2-165 and *O. ruminantium* GH1 was markedly lower than that of *A. muciniphila.*

## Discussion

Gut commensals are recognized as an important factor in maintaining host health. However, the role of the gut microbiome in Pb detoxification has not been well addressed. In this study, we observed that the intestinal absorption and tissue accumulation of Pb were exacerbated in mice lacking intestinal microbiota (Figure 1), suggesting that gut microorganisms can play a role in limiting the bioavailability of Pb.

The protective effects of gut microbiota against Pb absorption may be due to the following reasons. First, consistent with an earlier report (Reikvam et al., 2011), our results showed that the cecum is greatly enlarged in antibiotics-treated animals (Supplementary Figure S1C). This may prolong the retention of Pb in the gut and increase the absorption of this heavy metal. Second, it has been reported that compared with normal mice, gut commensal depletion by antibiotics increases gut permeability (Wu et al., 2014) and inhibits the expression of TJ proteins (Shi et al., 2018). Similar results were also observed in our acute Pb-exposure trial (Figure 2). Antibiotic treatment also elevates the expression of MT1 and MT2, which are metal-binding proteins with a high affinity for divalent cations crossing the apical membrane (Loumbourdis et al., 2007). This could in turn exacerbate the permeation of Pb through the gut barrier. Third, Pb undergoes enterohepatic circulation in the body and can be released into the gut from the liver via bile (Zhai et al., 2019). Antibiotic cocktail treatment decreases fecal BA excretion and leads to more effective BA conservation by strongly elevating the expression of the intestinal apical sodium-dependent bile salt transporter (Out et al., 2015), indicating elevated Pb reabsorption in the distal ileum and bioaccumulation in the liver. Finally, gut microbes such as *Lactobacillus*, *Bifidobacterium* (Teemu et al., 2008), and *Enterococcus* (Topcu and Bulat, 2010) were proved to bind and remove heavy metals via their surface compounds such as capsular polysaccharides and teichoic acids. This indicates that gut microorganisms may function as a biological barrier to directly compete with Pb absorption by the gut, thus limiting the bioavailability of this toxic metal.

Previous results indicated that among the entire gut microbiota population, some specific microbes, such as *Ruminiclostridium_9*, *Oscillibacter, Akkermansia* and others were extremely sensitive to Pb exposure (Zhai et al., 2017). We thus hypothesized that oral supplementation with these strains may be protective against Pb toxicity. From this study, we have shown that oral administration of *F. prausnitzii* and *O. ruminantium* had profound effects on relevant biomarkers – i.e., Pb content, gut barrier integrity, oxidation resistance, and the production of SCFA – showing that these bacteria significantly alleviated Pb-induced toxicity in mice, while *A. muciniphila* treatment failed to provide this protection.

The protective effects of *F. prausnitzii* treatment against chronic Pb exposure may be explained as follows. First, the increased gut permeability in different models is markedly associated with the reduced abundance of *F. prausnitzii* in feces of mice (Leclercq et al., 2013; Machiels et al., 2014), and the supplementation of *F. prausnitzii* can exert protective effects on the intestinal mucosal barrier (Laval et al., 2015). The mechanism may involve gene regulation controlling the intercellular barrier, the enhancement of SCFAs production, and benign interactions with gut microorganisms. Accordingly, our results showed that *F. prausnitzii* treatment strengthened the expression of TJ proteins such as ZO-1, occludin and claudin-1 in Pb-exposed mice (Figure 5). This was in line with an earlier finding that the expression levels of claudin-4 and F11r in the colon of mice were significantly increased after treatment with this bacterial strain (Laval et al., 2015). We also observed that *F. prausnitzii* treatment significantly increased the concentrations of SCFAs in colonic products (Figure 6). This is in accordance with a previous report showing the ability of *F. prausnitzii* to produce butyric acid or other SCFAs (Machiels et al., 2014). Upregulation of SCFAs, especially butyric acid, can reduce the pH of the intestinal lumen and trigger a number of signaling effects including the regulation of mucin synthesis and secretion (Paassen et al., 2009), further reinforcing the intestinal barrier (Wong et al., 2006). An earlier report provided direct *in vivo* evidence that *F. prausnitzii* was able to utilize acetate produced by *B. thetaiotaomicron* and that this symbiotic relationship exerted far-reaching and beneficial influences on the regulation of the bowel mucus barrier (Wrzosek et al., 2013). It is apparent that breaches in this physical barrier can aggravate the absorption of heavy metals, which indicates that enhancement of the biological barrier by *F. prausnitzii* treatment can prevent leakage and keep exogenous toxicants such as Pb out of the body, thus limiting the physical harm of Pb. Second, chronic Pb exposure directly induces the apoptosis of epithelial cells and marked local bowel inflammation, resulting in systemic disorders and elevated Pb absorption in the gut. Interestingly, levels of *F. prausnitzii* are inversely correlated with inflammatory markers such as IL-6 in low-grade inflammatory disease, indicating that this beneficial bacterium may play an inhibitory role in local intestinal inflammation and infection (Furet et al., 2010). Other studies have demonstrated that this normally abundant bacterium strongly induces the expression of microbial anti-inflammatory molecules (MAM) such as IL-10 in human and mouse dendritic cells and blocks NF-κB signals, ultimately supporting the homeostasis of the mucosal immune barrier (Hornef and Pabst, 2015; Rossi et al., 2016). This immunomodulatory effect of *F. prausnitzii* may effectively maintain the integrity of the epithelial barrier, thus minimizing Pb toxicity in the body. Third, a lowered abundance of fecal *F. prausnitzii* is observed in patients with inflammatory diseases such as infectious colitis and IBD (Sokol et al., 2009; Duboc et al., 2013), usually accompanied by an elevated levels of hepatic BAs and diminished levels of fecal unconjugated BAs. Consistent with the protective effects of *L. plantarum* CCFM8661 (Zhai et al., 2019), the fact that *F. prausnitzii* administration could facilitate fecal Pb excretion may be partly due to its role in regulating BA hepato-enteric circulation. *F. prausnitzii* treatment may inhibit BA re-uptake and promote the hepatic synthesis of BAs by repression of the enterohepatic FXR–FGF15 axis (Jia et al., 2018). This can limit the reflux of Pb from the gut to the liver via bile, thus giving rise to an increase in the biliary Pb output. In addition, the surface of Gram-positive bacteria contain negatively charged groups such as carboxyl, hydroxyl, and phosphate groups, and these can bind metals (Teemu et al., 2008). This may explain the observation that the supplementation of *F. prausnitzii*, a Gram-positive resident bacteria in the gut, significantly increased fecal Pb excretion due to its good Pb sequestration capability, and further mitigated chronic Pb burden for the mice when Pb was orally introduced (Figure 4). Recently, *F. prausnitzii* has been reported to provide full protection against arsenic toxicity in a murine model (Coryell et al., 2018), similar to our results.

Compared to *F. prausnitzii* treatment, the protective effects of *O. ruminantium* administration against Pb toxicity was less significant. *O. ruminantium* markedly reversed Pb-induced alterations in gene expression levels of TJ proteins, and the concentrations of total SCFAs and butyric acid (Figures 5, 6), which may be closely related to its capability of producing butyric acid (Lee et al., 2012). Recent studies have demonstrated that butyric acid, a major source of energy for intestinal epithelial cells, can increase the mucus-layer replenishment rate (Gaudier et al., 2004) and induce the differentiation of T_reg_/T_r__1_ cells via inhibition of histone deacetylation. This short chain fatty acid can also suppress inflammatory responses in the gut by downregulating the expression of INF-γ, TLR2, and TNF-α (Nicholas et al., 2013; Elce et al., 2017). Different inflammatory disorders such as IBD, enteric infections, and necrotizing enterocolitis (Lechuga and Ivanov, 2017) are usually manifested by increased permeability of the epithelial barrier. This suggests that the communication between the SCFAs induced by *O. ruminantium* and the immune response in intestinal mucosa may facilitate the repair of the intestinal barrier degradation caused by Pb exposure, thus limiting the uptake and toxicity of Pb in the host. However, this strain showed no obvious effect on renal and fecal Pb content (Figure 1), which may be because of its weaker Pb-binding capacity. Thus, the limited Pb-absorption ability of *O. ruminantium* means it cannot provide the same protection as *F. prausnitzii* in chronic Pb-exposed mice.

In contrast to both *F. prausnitzii* and *O. ruminantium* treatment, *A. muciniphila* treatment failed to ameliorate Pb toxicity (Figures 3–6). This may be closely connected with the reported opposing effects of *A. muciniphila* in regulating intestinal barrier function. Several studies have reported that *A. muciniphila* can restore mucus layer thickness and alleviate the intestinal inflammation reaction via pili-like proteins, and improve inflammation-induced loss of barrier integrity, thus reducing gut barrier disruption (Ottman et al., 2017; Chelakkot et al., 2018). In contrast, other experiments demonstrated that in DSS-treated or pathogen-infected colitis models, commensal *A. muciniphila* could drive alterations in mucosal constituents, disturb the homeostasis of the mucus layer and further exacerbate inflammation due to excessive degradation of mucin (Ganesh et al., 2013; Seregin et al., 2017). Supporting our results, a recent study showed that oral administration of *A. muciniphila* failed to exhibit protective effects against both acute and chronic cadmium toxicity (Feng et al., 2019). In this study, we also found that *A. muciniphila* ATCC BAA-835 was extremely sensitive to Pb (Table 5), which limits the viability of this strain when interacting with Pb in the gut and restricts its role in reducing Pb toxicity.

## Conclusion

Collectively, these results show that gut microbiota can limit the absorption and accumulation of Pb in host tissues. The oral administration of Pb-intolerant intestinal microbes such as *F. prausnitzii* and *O. ruminantium* significantly reduced Pb accumulation and alleviated Pb toxicity in mice, indicating that the modulation of gut microbiota may be important for the reduction of Pb toxicity burden in the host.

## Data Availability Statement

The raw data supporting the conclusions of this article will be made available by the authors, without undue reservation, to any qualified researcher.

## Ethics Statement

The animal study was reviewed and approved by the Ethics Committee of Jiangnan University, China.

## Author Contributions

QZ and WC designed the experiments. QZ, DQ, and YY carried out the experiments. DQ and SF developed the analysis tools. QZ and DQ wrote the manuscript. LY, FT, JZ, HZ, and WC revised the manuscript.

## Conflict of Interest

The authors declare that the research was conducted in the absence of any commercial or financial relationships that could be construed as a potential conflict of interest.
