# Supplementary material for: Oral Supplementation of Lead-Intolerant Intestinal Microbes Protects Against Lead (Pb) Toxicity in Mice
Source: Front Microbiol. 2020 Jan 22;10:3161. doi: 10.3389/fmicb.2019.03161 (PMC6987320; doi:10.3389/fmicb.2019.03161)
Supplement: Supplementary file 1 [file Table_1.docx]

Supplementary Material





**Supplementary Figure 1.** The effects of antibiotic treatment on depletion of gut commensals of mice (n=10). (A). Body weight, presented as percent of base line (= Day 0), of mice successfully depleted of cultivable fecal microbiota by gavage (VNMA) or gavaged with water in equivalent volume and frequency. (B). Cecal images from sham-treated (H_2_O), antibiotic-treated mice (bar, 1 cm). (C). Fecal matter was cultured aerobically and anaerobically. (D). Bacterial 16S DNA load (means ± SD) in fecal pellets. ***p* <0.01 and **p* <0.05 vs the control group; ## *p* <0.01 and #*p* <0.05 vs the Pb group.





**Supplementary Figure 2.** Representative photomicrographs of hepatic tissue of mice (H&E staining; magnifications, *×*200). (A) Control group; (B) Pb group; (C) Pb+*L. plantarum* group; (D) Pb+*O. ruminantium* group; (E) Pb+*A. muciniphila* group; (F) Pb+*F. prausnitzii* group.





**Supplementary Figure 3.** Representative photomicrographs of renal tissue of mice (H&E staining; magnifications, *×*200). (A) Control group; (B) Pb group; (C) Pb+*L. plantarum* group; (D) Pb+*O. ruminantium* group; (E) Pb+*A. muciniphila* group; (F) Pb+*F. prausnitzii* group.
