# Supplementary material for: Oral Supplementation of Lead-Intolerant Intestinal Microbes Protects Against Lead (Pb) Toxicity in Mice
Source: Front Microbiol. 2020 Jan 22;10:3161. doi: 10.3389/fmicb.2019.03161 (PMC6987320; doi:10.3389/fmicb.2019.03161)
Supplement: Supplementary file 2 [file Table_2.docx]

Supplementary Material

**Supplementary Table 1.** The effects of Pb-intolerant gut microbes on the values of haematogenic immunity paramaters (means ± SD) chronic Pb-exposed mice (n=10)

| Group | Neu#  (10^9^/L) | Lym#  (10^9^/L) | WBC  (10^9^/L) | Mon#  (10^9^/L) | Bas#  (10^9^/L) | Eos#  (10^9^/L) |
| --- | --- | --- | --- | --- | --- | --- |
| Control | 1.69±0.45 | 4.84±0.59 | 7.10±2.71 | 0.11±0.05 | 0.03±0.02 | 0.15±0.14 |
| Pb | 0.99±0.05 | 9.22±0.46* | 11.36±1.90 | 0.19±0.12 | 0.03±0.03 | 0.08±0.02 |
| Pb+*L. plantarum* | 1.30±0.09 | 7.81±1.38 | 10.30±1.30 | 0.13±0.10 | 0.03±0.01 | 0.11±0.04 |
| Pb+*O. ruminantium* | 1.59±0.32 | 8.57±3.32 | 11.44±3.27 | 0.26±0.11 | 0.04±0.04 | 0.23±0.12 |
| Pb+*A. muciniphila* | 1.29±0.26 | 8.26±1.31 | 9.62±1.72 | 0.18±0.09 | 0.01±0.01 | 0.10±0.04 |
| Pb+*F. prausnitzii* | 1.32±0.30 | 7.86±1.19 | 11.04±2.19 | 0.23±0.11 | 0.02±0.02 | 0.11±0.06 |

Note: **p* <0.05 vs the control group.

**Supplementary Table 2.** The effects of Pb-intolerant gut microbes on the values of basic hematological parameters (means ± SD) chronic Pb-exposed mice (n=10)

| Group | RBC (10^12^/L) | HGB (g/L) | MCH (pg) | MCHC (g/L) | RDW-CV (%) | RDW-SD (fL) | PLT (10^9^/L) |
| --- | --- | --- | --- | --- | --- | --- | --- |
| Control | 11.22±0.73 | 184±12 | 16.3±0.2 | 342±6 | 14.1±0.1 | 29.0±0.5 | 1285±276 |
| Pb | 9.17±1.32* | 148±20* | 14.3±0.8* | 338±13 | 14.3±0.6 | 26.2±1.1 | 1099±466 |
| Pb+*L. plantarum* | 10.56±1.12 | 171±3# | 14.9±0.3 | 349±7 | 14.2±0.6 | 28.1±1.7 | 1352±189 |
| Pb+*O. ruminantium* | 10.93±0.44 | 163±6# | 14.9±0.2 | 344±7 | 15.6±0.4 | 29.3±1.6 | 1507±301 |
| Pb+*A. muciniphila* | 10.30±1.03 | 160±10 | 14.9±0.2 | 344±8 | 14.7±0.4 | 27.5±1.3 | 1476±141 |
| Pb+*F. prausnitzii* | 10.60±1.05 | 166±10# | 15.1±0.2# | 348±9 | 14.8±0.8 | 27.1±1.3 | 1415±182 |

Note: **p* <0.05 vs the control group; #*p* < 0.05 vs the Pb group.
